# Construction of a ceRNA Network and a Prognostic lncRNA Signature associated with Vascular Invasion in Hepatocellular Carcinoma based on Weighted Gene Co-Expression Network Analysis

**DOI:** 10.7150/jca.57260

**Published:** 2021-05-05

**Authors:** Haisu Tao, Jiang Li, Junjie Liu, Tong Yuan, Erlei Zhang, Huifang Liang, Zhiyong Huang

**Affiliations:** 1 Hepatic Surgery Center, Tongji Medical College, Tongji Hospital, Huazhong University of Science and Technology, Wuhan, China.; 2 Hubei Key Laboratory of Hepato-Pancreato-Biliary Diseases, Wuhan, China.

**Keywords:** HCC, vascular invasion, ceRNA, prognostic signature, BBOX1-AS1

## Abstract

**Background:** Understanding risk factors for vascular invasion (VI) is crucial for assessing the risk of recurrence and overall prognosis of hepatocellular carcinoma (HCC). This study aimed to construct a prognostic long non-coding RNA (lncRNA) signature and a ceRNA Network associated with vascular invasion in HCC.

**Methods:** Differentially expressed genes (DEGs) of HCC patients associated with VI were identified by analyzing data from TCGA. Weighted gene co-expression network analysis (WGCNA) was used to identify associations between gene expression modules and clinical features. A VI-related prognostic lncRNA signature was then established using univariate, LASSO and multivariate Cox proportional hazards regression analyses. Based on the hub modules identified by the WGCNA, we constructed a VI-related lncRNA-miRNA-mRNA ceRNA network and screened hub lncRNAs for further research. Finally, we conducted *in vitro* and *in vivo* experiments to determine the biological roles of the identified hub gene BBOX1-AS1.

**Results:** The key module related to VI and OS was identified using WGCNA, after which a prognostic model consisting of eight lncRNAs was established, and verified using time-dependent receiver operating characteristic (ROC) curve analysis. BBOX1-AS1 was confirmed to be highly expressed in HCC tissues, and its expression was significantly correlated with a poor prognosis. Silencing BBOX1-AS1 *in vitro* significantly suppressed the proliferation, migration and invasion of HCC cells. *In vivo* experiments demonstrated that knocking down of BBOX1-AS1 could result in significant decrease of tumor volume and tumor weight.

**Conclusions:** The VI-related lncRNA signature established in this study can be used to predict the clinical outcomes of HCC patients. In addition, we constructed a VI-related lncRNA-miRNA-mRNA ceRNA network and demonstrated that BBOX1-AS1 might be a novel biomarker associated with VI in HCC.

## Introduction

HCC is the sixth most common malignancy and the fastest rising cause of cancer-related death globally 1. HCC has a high recurrence rate and poor survival, mainly due to its high invasiveness and metastatic potential 2-3. As a critical risk factor of HCC recurrence, vascular invasion is essential for tumor growth and makes it possible for aggressive tumor cells to leave the primary tumor and metastasize 4-5. VI can be divided into microscopic and macroscopic types, both of which are correlated with tumor recurrence and poor survival 6-7. Therefore, understanding the risk factors for VI is crucial for assessing the risk of tumor recurrence and metastasis.

Recent studies have demonstrated that some protein-coding genes as well as genes encoding non-translated RNAs contribute to the progression and metastasis of HCC by promoting VI. LOXL4 and FAM134B were recognized as oncogenes that promote the epithelial-to-mesenchymal transition (EMT) and tumor metastasis in HCC 8-9. In addition, some studies have shown that miRNAs and lncRNAs can regulate the development, proliferation, and apoptosis of HCC 10-11. Thus, many miRNAs and lncRNAs were identified as potential biomarkers for VI in HCC. MiR-218 and miR-345 can suppresses the metastasis of HCC by regulating the EMT 12-13, while lncRNA SBF2-AS1, UCID and ATB can promote the EMT and migration by acting as a ceRNA or regulating EMT-related genes expression in HCC 14-16. Several studies established and validated prognostic models based on VI-related miRNAs 5,17. However, there are still no reports of prognostic models for HCC based on VI-related lncRNAs.

Several studies confirmed that a lncRNA-miRNA-mRNA ceRNA network is related to the development of HCC 18,19. Moreover, researchers also constructed lncRNA-miRNA-mRNA ceRNA networks for HCC using public databases 20,21. However, there are still no reports on the construction of a ceRNA network for HCC using VI-related genes. Exploration of specific ceRNA elements associated with vascular invasion might facilitate more precise early detection, prevention or targeted therapeutic strategies. Therefore, the construction of a VI-related ceRNA network for HCC may reveal novel biomarkers associated with vascular invasion.

Using TCGA, we screened DEGs of HCC patients associated with vascular invasion. We also used WGCNA to identify the association between gene expression modules and clinical features. A VI-related prognostic lncRNA signature was then established using univariate, LASSO and multivariate Cox proportional hazards regression analyses, and verified using time-dependent ROC curve analysis. In addition, we constructed a VI-related lncRNA-miRNA-mRNA ceRNA network. We then analyzed the lncRNAs from the turquoise module of WGCNA and identified BBOX1-AS1 as a hub lncRNA for further research. Finally, the effects of the identified hub gene were validated *in vitro*.

## Materials and Methods

### Data retrieval and processing

Expression profiles for mRNAs, lncRNAs, and miRNAs, together with corresponding clinical information of liver hepatocellular carcinoma (LIHC) patients were manually downloaded from the TCGA data portal (https://portal.gdc.cancer.gov/). Using the human GENCODE database (https://www.gencodegenes.org/), we transformed the RNA sequence data into lncRNAs and mRNAs. The LIHC cohort contained 374 tumor samples and 50 normal samples. Since the data was derived from TCGA, no further approval was required from the Ethics Committee.

16 patients with macroscopic invasion and 93 patients with microvascular invasion were identified as patients with VI to further research. After normalization and filtering using the “edgeR” package in the R language, P -value < 0.05 and | logFC | > 1 were used for screening DEGs in HCC patients with VI versus those without VI. Then, the differentially expressed lncRNAs, miRNAs, and mRNAs meeting the criteria were displayed in volcano plots. A total of 342 patients with a follow-up time of ≥3 months were randomly divided into validation and training cohorts at a ratio of 3:7 for prognostic analysis, and the clinical features of patients in the two groups were similar.

### Constructing the ceRNA network

We used the miRcode database to predict interaction pairs between DElncRNAs and DEmiRNAs. The target mRNAs of miRNAs were retrieved from the miRDB, miRTarBase and TargetScan databases. To increase the reliability of the results, only the miRNA-mRNA pairs found in all 3 databases were selected as candidate genes for constructing the ceRNA network. The ceRNA network was constructed based on the DEmiRNA-DElncRNA and DEmiRNA-DEmRNA interaction pairs, and visualized using Cytoscape software (version 3.7.2; https://www.cytoscape.org/).

### Construction of a Weighted Gene Co-expression Network

Weighted gene co-expression network analysis was performed to predict the potential roles of the identified lncRNAs in the progression of HCC. The WGCNA R package was used to assess the relative importance of lncRNAs and their module membership. We first used paired Pearson correlations to evaluate the weighted co-expression relationships among the subjects from all datasets in the adjacency matrix. Then, a topological overlap matrix (TOM) similarity function was used to convert the matrix to a TOM. Subsequently, at least 50 co-expressed genes were aggregated into different modules using the dynamic tree cut method. Finally, P < 0.05 was used as the statistically significant standard to screen important gene modules. The module-trait relationships showed that the turquoise module was most significantly related to VI. Therefore, the turquoise module was selected for subsequent analyses to explore key genes.

### Construction and Validation of a Prognostic Model

In order to find the most relevant prognostic genes, the DElncRNAs from the turquoise module were used to construct a prognostic risk signature via univariate Cox regression. LncRNA expression differences were considered statistically significant at P < 0.05. For the training group, the screened lncRNAs were further selected and validated through LASSO regression using the “glmnet” R package. Multivariate Cox regression analysis was subsequently used to assess the contribution of each lncRNA and further select the best model using a retrograde stepwise method. A risk score was calculated based on a linear combination of the lncRNA expression level and a multiplied regression coefficient (β) with the following formula: the risk score=(β* expression level of ×1)+(β* expression level of ×2)+(β* expression level of ×3)+(β* expression level of…×n). The prognostic value of the model was examined through Kaplan-Meier survival curve and the time-dependent ROC curve analysis in the training set, testing set and entire set. Subsequently, univariate and multivariate Cox regression analyses were used to evaluate whether the prognostic signature could be an independent prognostic factor when combined with other clinical information.

### Gene Set Enrichment Analyses and CIBERSORT

To explore the potential molecular mechanisms underlying our constructed prognostic gene signature, GSEA was performed using the Molecular Signatures Database (MSigDB) of KEGG gene sets (c2.cp.kegg.v6.2.symbols) and immunological signature gene sets (c7.all.v7.0.symbols.gmt). Hits with a P < 0.05 and false discovery rate (FDR) q < 0.25 were considered statistically significant. Normalized gene expression data were used to infer the relative proportions of 22 types of infiltrating immune cells using the CIBERSORT algorithm. The association of the immune-related lncRNA signature with 22 infiltrating immune cells types was analyzed to explore whether this immune-related lncRNA signature may play a crucial role in immune infiltration in HCC.

### Tissue Specimens, Cell Culture and Transfection

A total of 43 paired freshly frozen HCC and corresponding noncancerous tissues were obtained from patients who underwent liver resection at the Hepatic Surgery Center of Tongji Hospital and were used for qRT-PCR. This study was approved by the Medical Ethics Committee of Tongji Hospital and conducted in accordance with the Declaration of Helsinki. The HCC cell line MHCC-97H [97H] was purchased from the China Center for Type Culture Collection (CCTCC, Wuhan, China). The cell lines were cultured in Dulbecco's modified Eagle's medium (Invitrogen) supplemented with 10% fetal bovine serum (Gibco, Grand Island, NY) and incubated in a humidified atmosphere comprising 5% CO_2_ at 37 °C. The small interfering RNAs (siRNAs) and si-control were purchased from RiboBio (Guangzhou, China). Lipofectamine 3000 (Invitrogen, Carlsbad, CA, USA) was used for transient transfection according to the manufacturer's protocol. To conduct animal experiments, MHCC-97H cells were transfected with the specific short hairpin RNAs to BBOX1-AS1 (sh-BBOX1-AS1). SiRNA target sequences and sh-BBOX1-AS1 sequence are listed in Additional file 1: Table S1.

### RNA Isolation and qRT-PCR Analysis

TRIzol reagent (Invitrogen) was used to extract the total RNA from tissues and cells according to the manufacturer's protocol. The reverse transcription was conducted using a reverse-transcription system kit (Takara Bio, Dalian, China). The relative RNA expression levels were determined by qRT-PCR on a CFX Connect™ Real-Time PCR Detection System (Bio-Rad, Hercules, CA, USA) using the SYBR Green SuperMix kit (Takara Bio). All gene expression levels were normalized to that of the housekeeping gene glyceraldehyde-3-phosphate dehydrogenase (GAPDH). The primer sequences are listed in Additional file 1: Table S2.

### Transwell and Wound Healing Assays

Cell migration and invasion assays were performed in 24-well Transwell plates (Corning, MA, USA), according to the manufacturer's protocol. Tumor cells in serum-free medium were placed in the upper chamber and culture medium containing 10% FBS was placed in the lower chamber. We used an upper chamber that had been coated with Matrigel (2 mg/ml) for the invasion assay, and an untreated upper chamber for the migration assay. Additionally, 97H cells were cultured in 6-well-plates and scraped with a 200-μl pipette tip. The cells were cultured in DMEM without FBS. Cell migration was recorded under a phase-contrast microscope with white light at 0 and 48 h after scratching generation.

### Cell Proliferation Assay

The proliferation rate of cells was measured using the CCK-8 assay Kit (CCK-8, Beyotime Institute of Biotechnology, Shanghai, China). Cells were seeded into 96-well plates at the indicated density and cultured in complete medium. The absorbance was measured at 450 nM using a SpectraMax 250 spectrophotometer (Molecular Devices, USA). The EdU assay (RiboBio, Guangzhou, China) was used to determine the cell proliferation capacity. The 97H cells were seeded into 96-well plates at a density of 1×10^4^ cells per well and cultured overnight. All operations were performed based on the kit instructions. Fluorescence microscopy was used to observe and analyze the number of EdU-positive cells.

### Colony formation Assay and Cell Cycle Analysis

The colony formation assay and cell cycle analysis were performed as described previously 22.

### Animal Studies

4-week-old male BALB/c mice were obtained from the HFK BioScience Corporation (Beijing, China). Animal studies were approved by the Ethics Committee of Tongji Hospital, Huazhong University of Science and Technology. 97H cells that were transfected with sh-BBOX1-AS1 or sh-NC were subcutaneously injected into the left and right back of nude mice, respectively. Tumors were measured every 3 days and harvested after 18 days of treatment.

### Statistical analysis

The GraphPad prism 7.0 software and R software (version 3.6.2) were used for picture editing. Experimental data were manifested as mean ± SD. Kaplan-Meier method and the log-rank test were used to determine differences in survival rates. Survival data were also evaluated using Cox regression model. Student's t test and one-way ANOVA were utilized for the group difference analysis. Unless otherwise noted, P < 0.05 was considered to be significant.

## Results

### Differentially expressed mRNAs, miRNAs and lncRNAs

**Figure 1:**
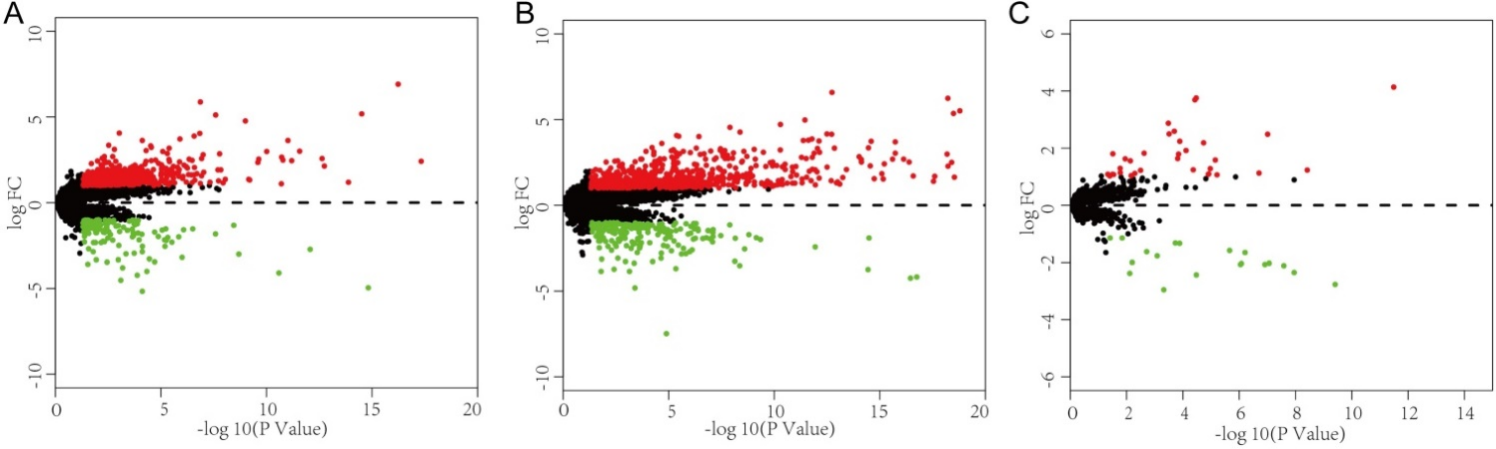
** Identification of differentially expressed genes and construction of a VI-related ceRNA network. (A-C)** Volcano plot of the DElncRNAs, DEmRNAs and DEmiRNAs. The red dots are defined as upregulated genes and green dots are defined as downregulated genes. Black dots represent no differentially expressed genes.

This study investigated the expression levels of RNAs in HCC samples from the TCGA database to identify DEGs may be related to VI in HCC patients. The 315 HCC patients who had clinical information on vascular invasion were separated into two groups, encompassing 109 patients with VI and 206 patients without VI. Differential expression analysis between the VI group and non-VI group (p < 0.05 and |log FC|>1) identified 592 DElncRNAs (457 upregulated, 135 downregulated), 53 DEmiRNAs (34 upregulated, 19 downregulated), and 893 DEmRNAs (695 upregulated, 198 downregulated) (Fig. 1).

### Construction of a VI-related ceRNA network for HCC

**Figure 2:**
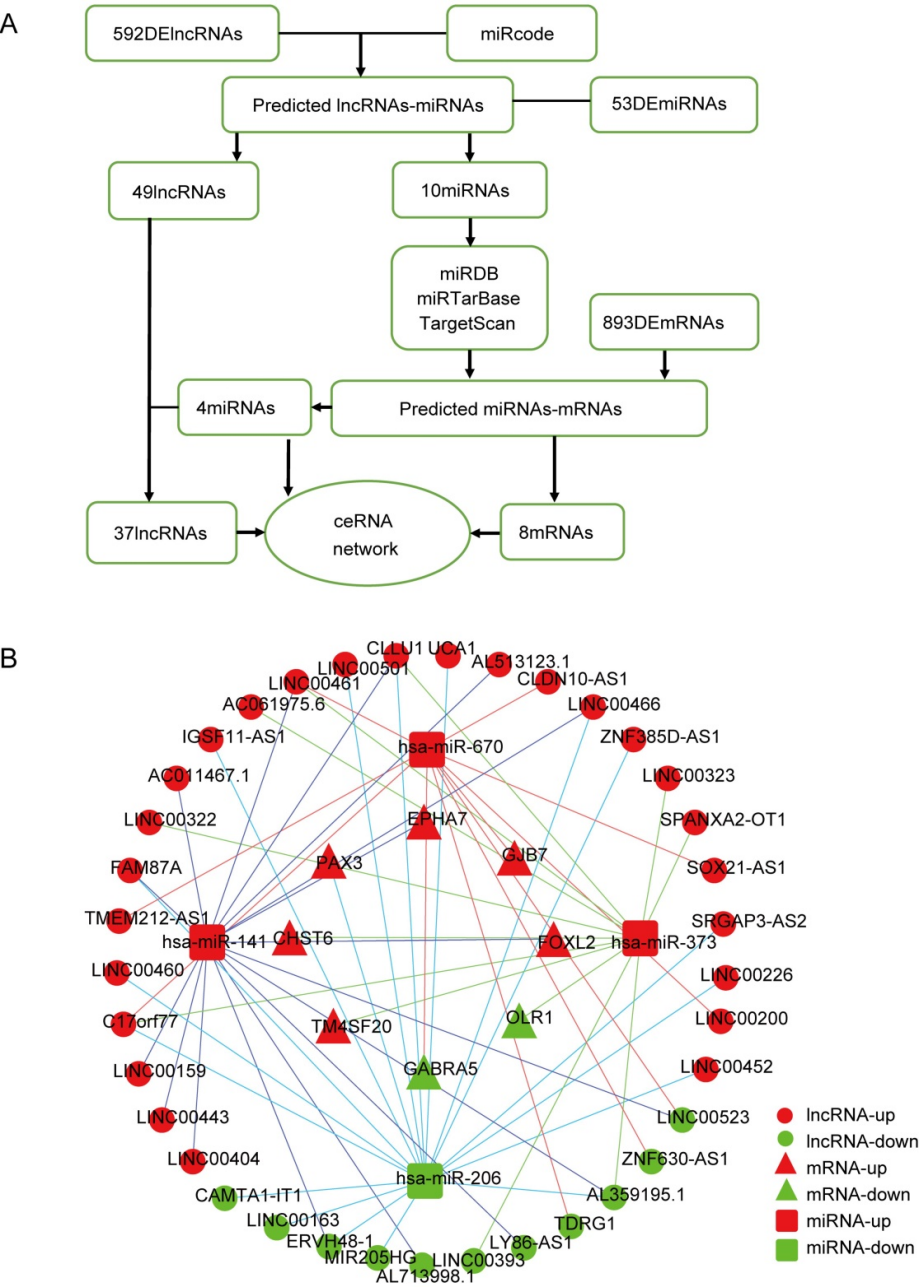
** Construction of a VI-related ceRNA network. (A)** Flow chart of ceRNA network construction. **(B)** VI-related ceRNA network. Red represents upregulated genes, green represents downregulated genes. Balls represent lncRNAs, squares represent miRNAs and triangles represent mRNAs.

To better understand the interaction network of lncRNAs, miRNAs and mRNAs related to vascular invasion in HCC, we constructed a lncRNA-miRNA-mRNA ceRNA regulatory network based on the identified DEGs. As shown in Fig. 2A, 10 DEmiRNAs were found to interact with 49 DElncRNAs using the miRcode database. Then, we searched for DEmRNAs based on the 10 DEmiRNAs in three databases (miRDB, miRTarBase, and TargetScan), and miRNA-mRNA interaction pairs encompassing 10 DEmiRNAs and 434 mRNAs were confirmed. The 434 targeted mRNAs were further intersected with the 893 retrieved DEmRNAs, and 8 DEmRNAs were found to be involved in the ceRNA regulatory network (Fig. 2B). After removing the remaining 6 DEmiRNAs and the corresponding lncRNAs, a total of 37 DElncRNAs, 4 DEmiRNAs, and 8 DEmRNAs were used to establish a ceRNA network (Fig. 2B).

### WGCNA and Identification of Key Modules Related to VI and OS

**Figure 3:**
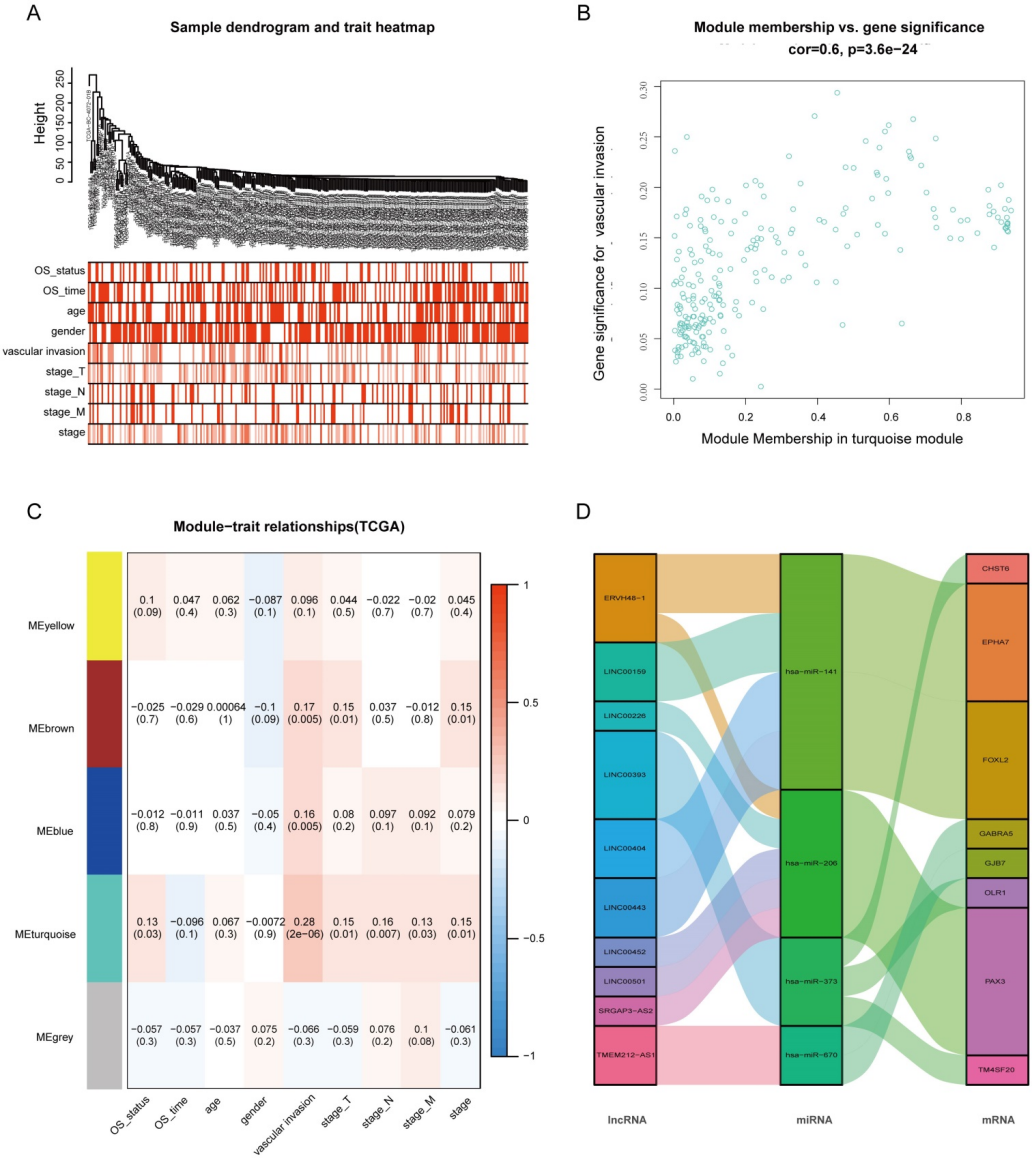
** Weighted gene co-expression network analysis (WGCNA). (A)** Sample dendrogram and trait heatmap. **(B)** Correlation between gene modules and clinical traits. **(C)** Scatter plot of genes in the turquoise module. **(D)** Sankey diagram for the VI-related hub ceRNA network of HCC. Left bar: lncRNA; middle bar: miRNA; right bar: mRNA.

After extracting the FPKM value expression matrix of the lncRNAs, a total of 592 DElncRNAs were selected and subjected to WGCNA, which was used to construct gene co-expression modules that assign these genes to different modules in cluster dendrograms (Fig. 3A). A plot of module membership vs. gene significance of the five HCC-related modules is shown in Figure 3B. The turquoise module not only exhibited the highest correlation with VI (cor = 0.28, P = 2e-06) but also showed significant module membership relevance to OS status (cor = 0.13, P = 0.03). Thus, the turquoise module was considered as the most important module related to VI and the prognosis of HCC (Fig. 3C).

The 37 DElncRNAs in the constructed ceRNA network were intersected with the 233 lncRNAs from the turquoise module, and 10 intersected lncRNAs were identified as the most VI-relevant lncRNAs. Based on the relationships in the abovementioned ceRNA network, we constructed a VI-related hub ceRNA network consisting of 8 mRNAs, 4 miRNAs, and 10 lncRNAs (Fig. 3D).

### Confirmation of the Prognostic lncRNA Signature

**Figure 4:**
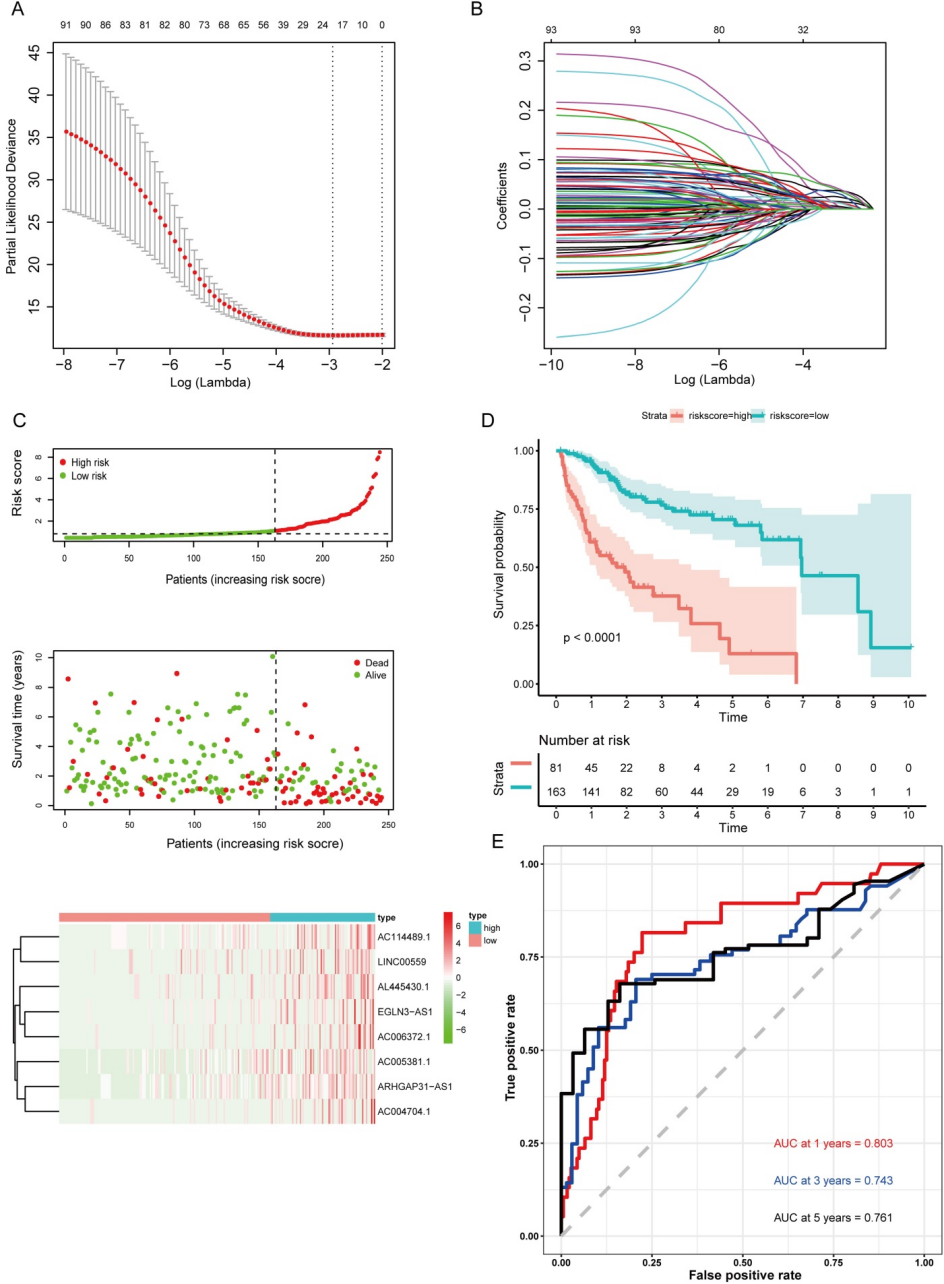
** Construction of the prognostic model in the training group. (A)** LASSO coefficient profiles for significantly correlated lncRNAs in univariate Cox regression analysis. **(B)** Cross-validation for selecting the optimal lambda value for the LASSO model. **(C)** Distribution of risk scores, survival status, and gene expression patterns of patients in high- and low-risk groups. **(D)** Kaplan-Meier survival curves of the low- and high-risk groups. **(E)** Time-dependent ROC curve analyses of low- and high-risk groups.

The 233 genes in the turquoise module were analyzed using univariate Cox regression and the results showed that a total of 93 DElncRNAs were significantly correlated with OS (P < 0.05). Next, LASSO regression was employed to verify additional variables in the training cohort (Fig. 4A,B). To identify potential prognostic markers among the survival-related variables, K-M survival analyses and log-rank tests were performed for each gene to evaluate the 19 lncRNAs. Based on 16 lncRNA that were significantly correlated with overall survival (Table S3), multivariate Cox regression analyses was applied to select potential prognostic lncRNAs, and their contributions were weighted by their relative coefficients. The final risk score formula was as follows: risk score = (0.2190 × expression level of EGLN3-AS1) + (0.3118 × expression level of AC114489.1) + (0.2786 × expression level of ARHGAP31-AS1) + (0.2622 × expression level of AC006372.1) + (0.2261 × expression level of AC004704.1) + (0.4013 × expression level of AL445430.1) + (0.2268 × expression level of LINC00559) + (0.1678 × expression level of AC005381.1). The lncRNA risk score for clinical prognosis in the training set was determined using the risk prediction formula.

### Validation of the Prognostic lncRNA Signature

The risk score was computed for every case, and all cases were classified as low- or high-risk based on the optimal cutoff value of the risk score derived using the survminer R package. Survival curves for the high-risk group were significantly different from the low-risk group, as shown in the Kaplan-Meier plots (Fig. 4C). In the training set, cases with high risk scores had shorter OS than low-risk cases (P<0.0001) (Fig. 4D). Additionally, time-dependent ROC curves were utilized to assess the prognostic performance of the lncRNA biomarkers. The AUC values of the ROC curve at 1, 3, and 5 years were 0.803, 0.743, and 0.761, respectively (Fig. 4E).

**Figure 5:**
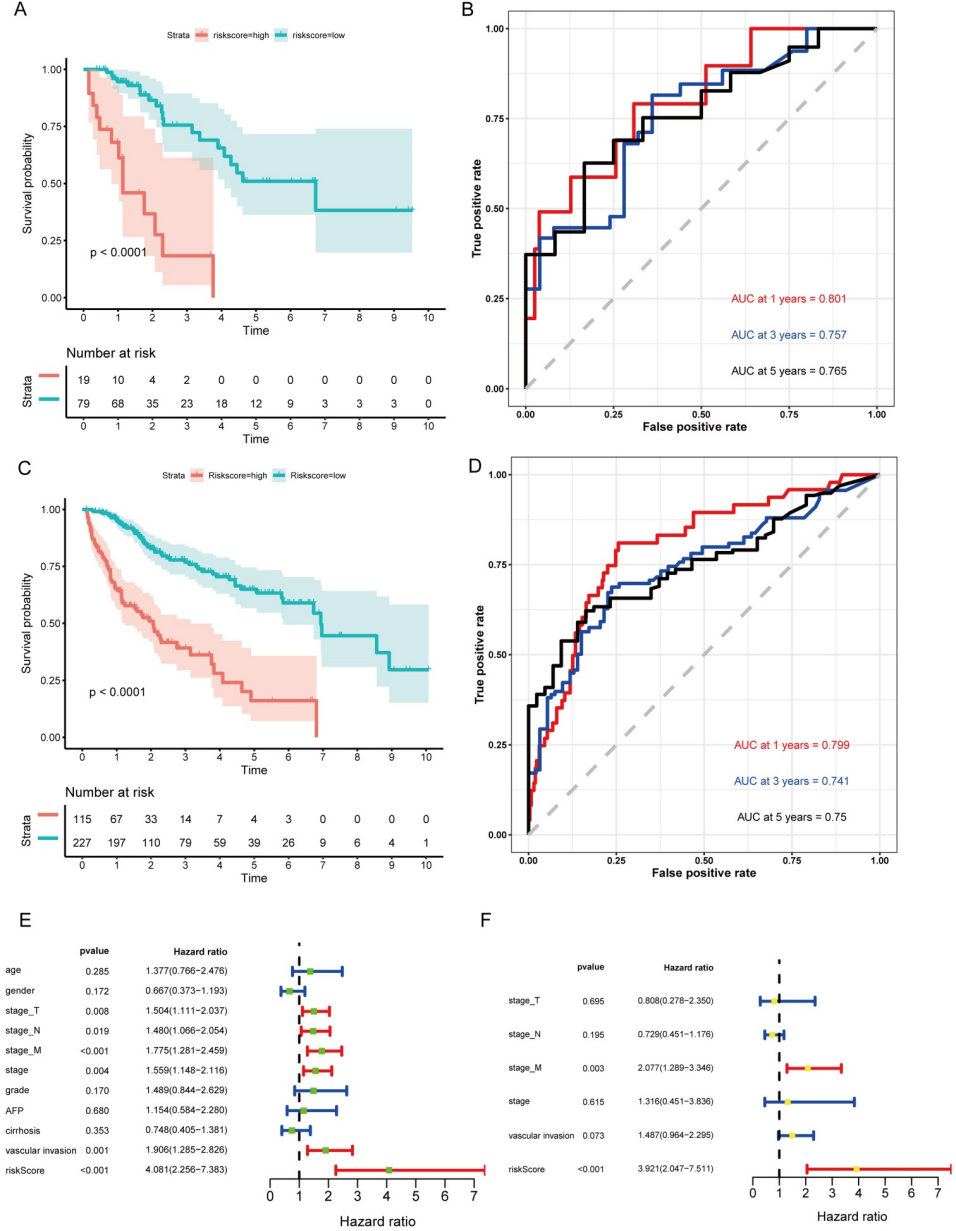
** Validation of the prognostic model in the validation cohort and the entire cohort. (A)** Kaplan-Meier survival curve for the validation cohort. **(B)** Time-dependent ROC curve analysis for the validation cohort. **(C)** Kaplan-Meier survival curve for the entire cohort. **(D)** Time-dependent ROC curves analysis for the entire cohort. **(E)** Univariate analysis results for the entire cohort. **(F)** Multivariate analysis results for the entire cohort.

The formula was further used in the validation cohort and the entire cohort to verify the prognostic significance. Figure 5A,C shows the Kaplan-Meier curves for the low- and high-risk groups in the validation cohort and the entire set. The OS of patients benefited from a low risk score in the validation cohort (P=0.00019) and the entire set (P<0.0001). The ROC curve analysis in the validation set showed AUC values at 1, 3, and 5 years of 0.801, 0.757, and 0.765, respectively (Fig. 5B). Finally, the ROC curve analysis for the entire set was also performed and the AUC values at 1, 3, and 5 years were 0.799, 0.741, and 0.75 (Fig. 5D).

In addition, the hazard ratio (HR) for the risk score following univariate Cox proportional hazards regression was 4.081 (95% confidence interval [CI]: 2.256-7.383; Fig. 5E). Consistent results were obtained through multivariate Cox proportional hazards regression (HR = 3.921, 95% CI: 2.047-7.511) adjusted for clinical covariates (Fig. 5F).

Therefore, all the results suggested that the VI-related lncRNA signature has good sensitivity and specificity for predicting the clinical outcomes of HCC patients.

### Functional Analysis of the Prognostic lncRNA Signature

**Figure 6:**
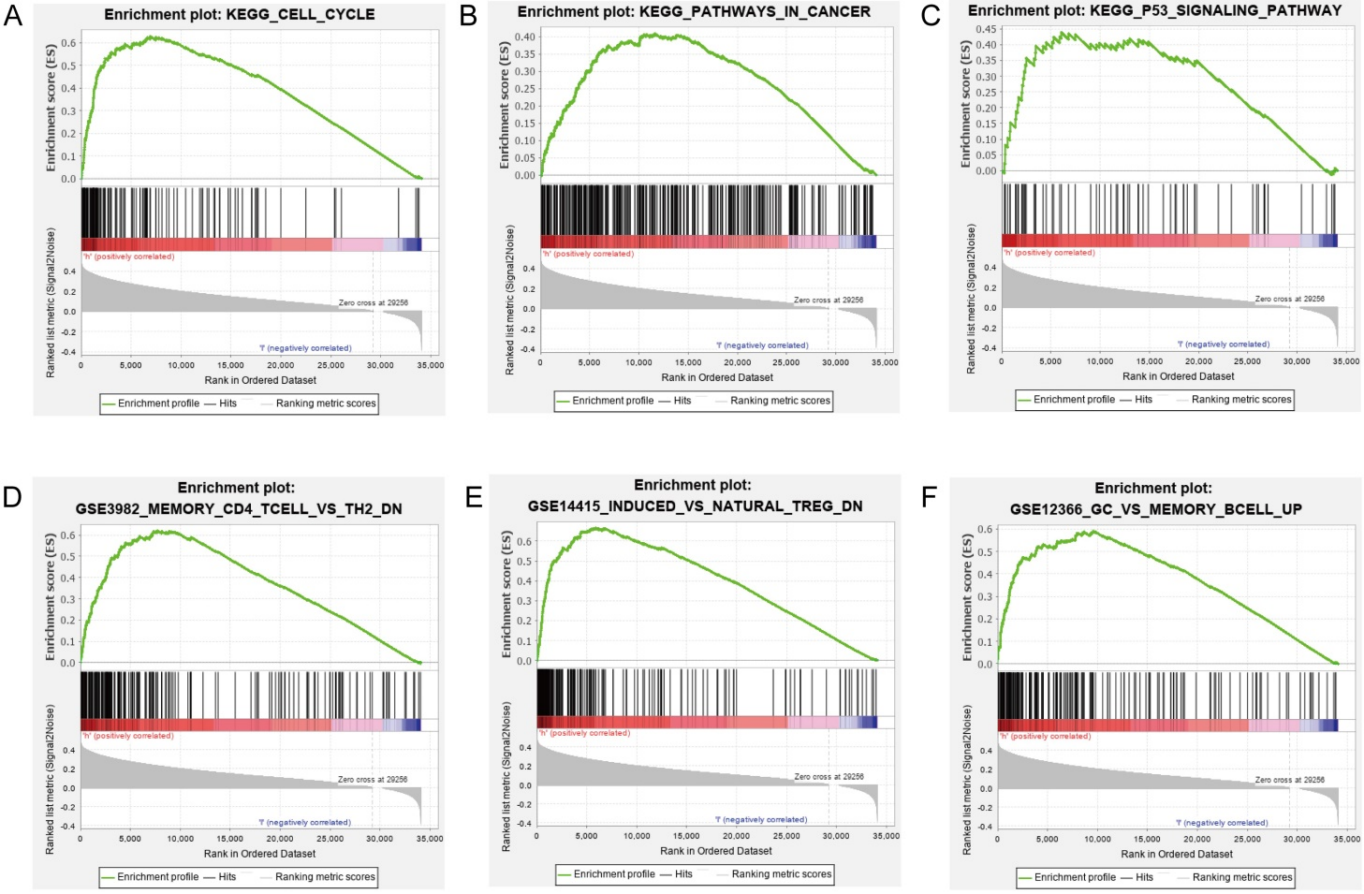
** GSEA of the biological pathways related to the lncRNA model. (A-C)** The significantly enriched KEGG pathways in the high-risk group. **(D-F)** The significantly enriched immunological signatures in the high-risk group.

Our results suggested that the group scoring high in the lncRNA signature showed significant enrichment in 39 pathways including cell cycle, DNA replication, metabolism, pathways in cancer, NOTCH signaling pathway, mTOR signaling pathway, and P53 signaling pathway (Figs. 6A-C). Elucidating the biological functions of the eight lncRNAs would help us gain insights into the underlying molecular mechanisms related to tumor initiation and progression. In the high-risk group, many immunological signatures were enriched, such as memory CD4^+^ T cells versus TH2 cells down, induced Treg cells versus natural Treg cells down, germinal center (GC) B cells versus memory B cells up, etc. (Figs. 6D-F). These findings suggested that the prognostic lncRNA signature is related to immune regulation.

**Figure 7:**
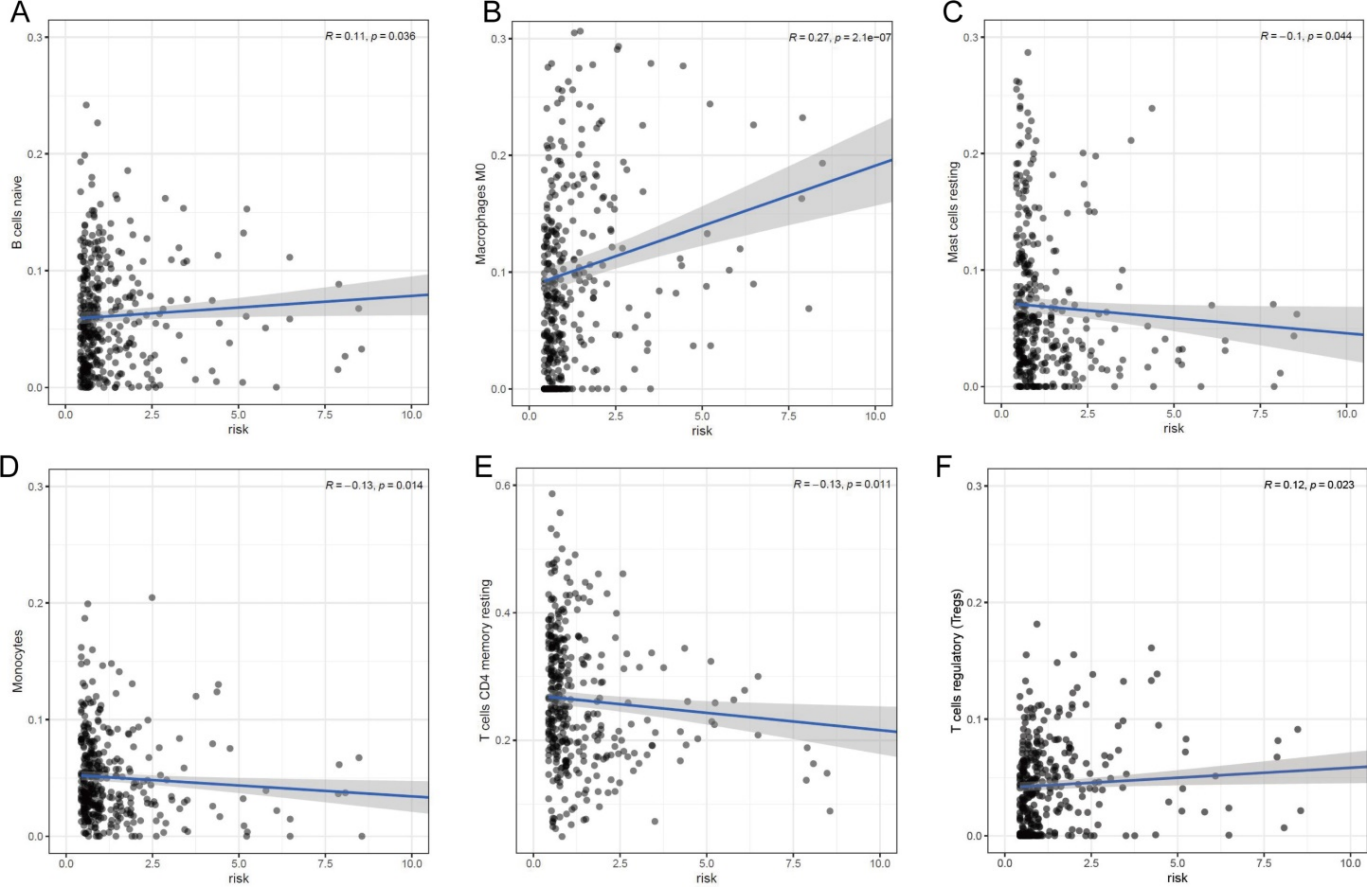
** Association between the lncRNA signature and tumor infiltration by different immune cell subsets. (A)** Correlation between the signature and infiltration by naïve memory B cells. **(B)** Correlation between the signature and M0 macrophages. **(C)** Correlation between the signature and regulatory T cells (Tregs). **(D)** Correlation between the signature and resting mast cells. **(E)** Correlation between the signature and monocytes. **(F)** Correlation between the signature and resting CD4^+^ memory T cells.

Our prognostic lncRNA signature significant positive correlations with tumor infiltration by naïve B cells (r =0.11; p = 0.036), M0 macrophages (r =0.27; p < 0.001), and regulatory T cells (Tregs) (r =0.12; p = 0.023), as well as significant negative correlations with tumor infiltration by resting mast cells (r =-0.1; p = 0.044), monocytes (r =-0.13; p = 0.014), and resting CD4^+^ memory T cells (r =-0.13; p = 0.011) (Fig. 7). These findings suggest that the prognostic lncRNA signature is associated with immune cell infiltration in HCC, which was consistent with the analysis of immunological signatures.

### Identification of Hub lncRNAs Related to VI and OS

Based on the criteria P < 0.05 and |LogFC| > 1, a total of 3752 DElncRNAs between HCC tumor samples and adjacent normal tissues were identified in the TCGA-LIHC database. Then, the 233 genes in the turquoise module were intersected with the 3752 DElncRNAs and 131 overlapping lncRNAs were identified (Fig. S1). We conducted univariate Cox regression analysis on the 131 overlapping lncRNAs and identified 62 genes significantly correlated with the OS of HCC patients (Table S4). Next, the top genes with low P-values were identified as hub genes. The most highly ranked hub gene, HOXA11-AS, was already recognized as an oncogene of HCC in previous studies. Therefore, the second most significant hub gene, BBOX1-AS1, whose relationship with HCC was not studied in detail, was chosen for more in-depth analysis.

### The expression and function of BBOX1-AS1 in TCGA

**Figure 8:**
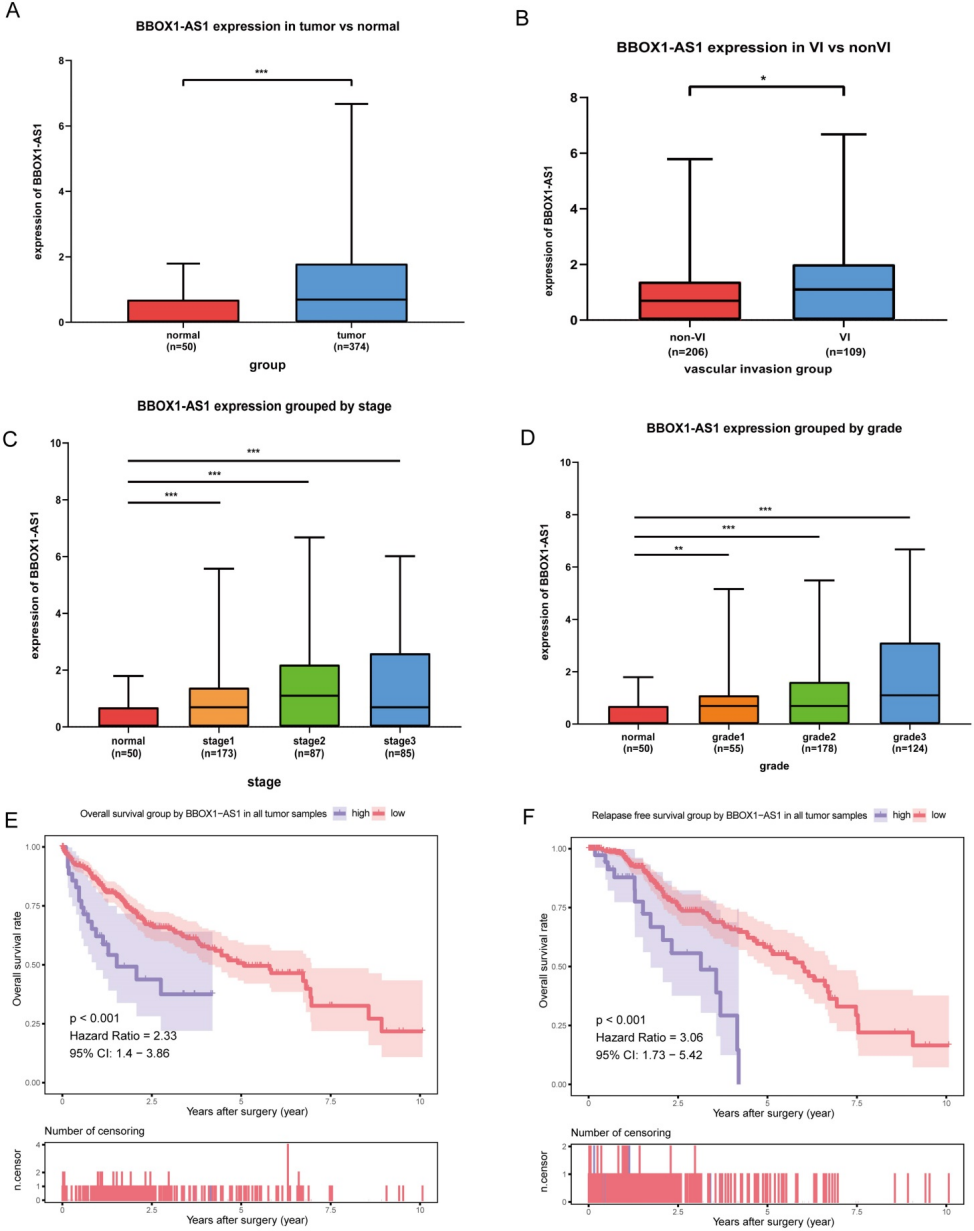
** The expression and function of BBOX1-AS1 in TCGA. (A)** The mRNA expression levels of BBOX1-AS1 were significantly higher in cancer tissues than in paired normal tissues. **(B)** The expression level of BBOX1-AS1 in patients with VI was higher than in patients without VI. **(C)** The expression level of BBOX1-AS1 was positively correlated with tumor stage in HCC patients. **(D)** The expression level of BBOX1-AS1 was positively correlated with tumor grade in HCC patients. **(E)** Overall survival analysis stratified by BBOX1-AS1 expresison based on TCGA data. **(F)** Disease free survival analysis stratified by BBOX1-AS1 expression based on TCGA data.

As shown in the boxplot (Fig. 8A), BBOX1-AS1 was found to be overexpressed in cancerous tissues compared with normal tissues in the TCGA LIHC dataset. Consequently, we decided to further analyze the expression of BBOX1-AS1 in HCC tissues from patients with VI and patients without VI, which revealed that BBOX1-AS1 was overexpressed in patients with VI (Fig. 8B). Moreover, we analyzed the relationship between BBOX1-AS1 expression and the pathological tumor grade or stage and found that the expression level of BBOX1-AS1 was positively correlated with both tumor grade and stage in HCC patients. These results indicated high BBOX1-AS1 expression was likely directly associated with poor clinical outcomes (Figs. 8C and D). To explore the impact of BBOX1-AS1 expression on the prognosis of patients, we performed a survival analysis and found that patients with high BBOX1-AS1 expression had shorter overall survival and relapse-free survival (Figs. 8E and F).

### Exploring the role of BBOX1-AS1 in HCC

**Figure 9:**
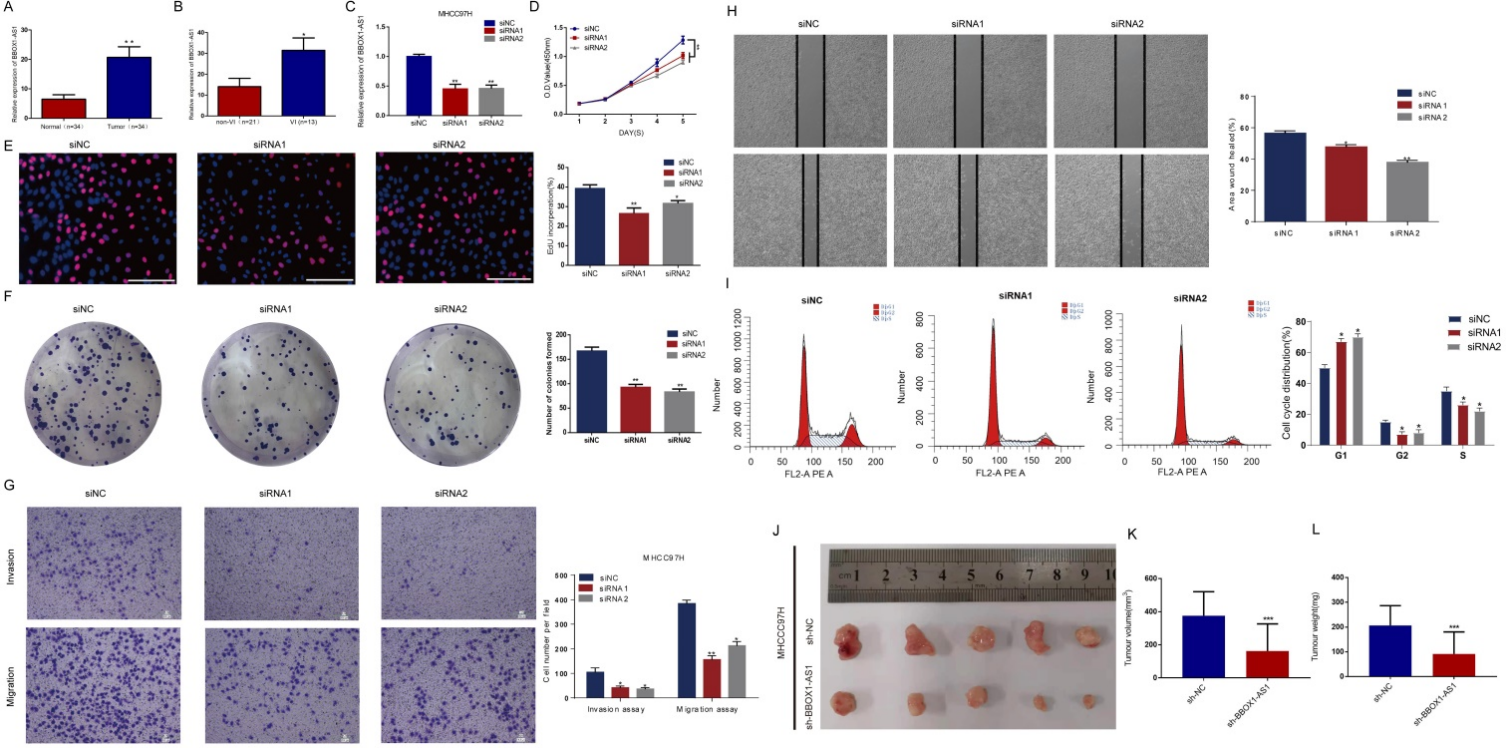
** Exploring the role of BBOX1-AS1 in HCC samples,cell culture, and *in vivo* study. (A)** BBOX1-AS1 was overexpressed in cancerous tissues compared with normal tissues in the Tongji cohorts. **(B)** BBOX1-AS1 was overexpressed in patients with VI compared with patients without VI in the Tongji cohorts. **(C)** Transfection efficacy of siRNAs in 97H cells. **(D)** The CCK-8 assay showed decreased proliferation after siRNA transfection. **(E)** The EdU assay showed decreased proliferation after siRNA transfection. **(F)** The colony formation assay showed decreased colony formation after siRNA transfection. **(G)** Transwell assays were used to detect HCC invasion and migration. **(H)** Wound healing assay. **(I)** Cell cycle was arrested in G0/G1 phase after transfection. **(K-L)** Tumour volume and tumour weight were analysed after transfected 97H cells were injected subcutaneously into nude mice (*p<0.05;**p<0.01; ***p<0.001).

Consistent with the results described above, BBOX1-AS1 was also overexpressed in cancerous tissues from HCC patients with VI in the Tongji cohorts (Figs. 9A and B). To further test its effects in tumor cells, *in vitro* study was carried out. The transfection efficiency of the cells was evaluated by qRT-PCR (Fig. 9C). To elucidate the proliferation rate of HCC cells after BBOX1-AS1 silencing, CCK-8 and EdU assays were performed. The CCK-8 assay yielded lower absorbance in HCC cells with BBOX1-AS1 silence at days 4 and 5 (Fig. 9D). The EdU assay also suggested a decrease of the proliferation ability in HCC cells with BBOX1-AS1 silence (Fig. 9E). Moreover, the colony formation assay also indicated that BBOX1-AS1 silencing significantly suppressed the growth of HCC cells (Fig. 9F). To further confirm the role of BBOX1-AS1 in invasion and migration, transwell assays were performed and the results showed that BBOX1-AS1 silencing significantly suppressed the invasion and migration rates of HCC cells (Fig. 9G). Accordingly, BBOX1-AS1 silencing also significantly repressed wound healing in HCC cells (Fig. 9H). The potential influence of BBOX1-AS1 on cell cycle was also analyzed in 97H cells, and the results revealed that the cell cycle was arrested in the G0/G1 phase following BBOX1-AS1 silencing (Fig. 9I). Consistent with *in vitro* observations, knocking down of BBOX1-AS1 resulted in significant decrease of tumor volume and tumor weight (Figs. 9 K-L).

## Discussion

As a well-known factor contributing to tumor recurrence and poor prognosis, VI is essential for tumor growth and makes it possible for aggressive cancer cells to leave the primary tumor for distant metastatic sites 5,17. Although there is limited information on the underlying molecular mechanisms that modulate VI, recent genomic analyses have demonstrated that unique genes and ncRNAs play important roles in metastasis 23. The advancement of high-throughput technologies has enabled the comprehensive analysis of gene expression and its incorporation into routine clinical prognosis for HCC patients.

Various studies have demonstrated that lncRNAs can be important prognostic factors for clinical outcomes because their dysregulation promotes the EMT, invasion and migration of HCC. For example, the lncRNA SNHG3 fas found to induce the EMT and sorafenib resistance by modulating the miR-128/CD151 pathway in HCC. Another study found that the lncRNA DANCR is associated with the EMT, leading to tumor recurrence and metastasis 24,25. Accorindlgy, several researchers developed prognostic lncRNA signatures for HCC 26,27. However, to our best knowledge, there is no prognostic model based on VI-related lncRNAs for HCC. In this study, we used the data of HCC patients available in TCGA to identify differentially expressed lncRNAs between patients with and without VI, which may be closely associated with patient survival. Different from previous publications, this study therefore aimed to predict the survival of HCC patients using a VI-related lncRNA signature. VI-associated DElncRNAs were comprehensively screened using WGCNA and univariate Cox analysis. Then, LASSO regression was applied to the lncRNA data from TCGA, and 19 lncRNAs were filtered out, after which Kaplan-Meier and multiple Cox regression analyses were utilized to construct the prognostic model. Finally, after the construction of the model, Kaplan-Meier analysis and time-dependent ROC curves were employed to confirm the prognostic significance of the lncRNA signature. Further validation was carried out in both the validation cohort and the entire cohort. These results demonstrate that the eight‐lncRNA signature is useful for predicting VI in HCC tumor tissues, and is therefore useful for clinical prognosis.

GSEA indicated that the lncRNAs from our prognostic signature are mainly involved in the cell cycle, DNA replication, metabolism and some canonical pathways related to HCC initiation and development. These GSEA findings provide new evidence that lncRNAs whose biological functions have not been reported to date could potentially serve as prognostic biomarkers for HCC. Nonetheless, these results should be validated in future studies, and the underlying molecular mechanisms should also be investigated.

Some lncRNAs can control tumor immunity, through mechanisms such as regulation of the tumor microenvironment (TME), immune cell infiltration, and immune cell differentiation. Several recent papers have shown that multiple lncRNAs may act as mediators of the function of dendritic cells (DCs), T cells, B cells, and immune tolerance. These lncRNAs include lincRNA-EPS in macrophages, the lncRNAs Smad3 and BLACAT1 in T cells, as well as the lncRNA MALAT1 in B cells 28-31. In this study, we also found that our ncRNA signature was correlated with HCC tumor infiltration by B cells, M0 macrophages, Tregs, resting mast cells, monocytes and resting CD4^+^ memory T cells. These results were consistent with analyses of the immunological signature of HCC, which suggested that the immune-related lncRNA signature may play a role in immune infiltration and immune regulation of HCC.

The analysis of public databases and construction of a comprehensive ceRNA regulatory network can reveal novel biomarkers associated with the tumorigenesis and development of HCC. Several recent studies analyzed the ceRNA network of HCC 21,32. However, there are no reports of a ceRNA network constructed based on VI. In this study, we identified differentially expressed lncRNAs, miRNAs and mRNAs between HCC patients with and without VI, which were then applied to construct an lncRNA-miRNA-mRNA ceRNA network. To the best of our knowledge, this is the first study to investigate the specific ceRNA network of VI-related DEGs in HCC. Further exploration of specific ceRNA elements associated with VI might facilitate more precise early detection, prevention or targeted therapeutic strategies.

In addition, we analyzed the lncRNAs of the turquoise module in WGCNA and screened hub lncRNAs for further research. BBOX1-AS1 is one of the best-studied oncogenic lncRNAs, but its role in HCC is still incompletely understood. In recent studies, BBOX1-AS1 was identified as an oncogene that is closely associated with various cancers. For example, Liu et al. found that BBOX1-AS1 contributes to colorectal cancer progression by sponging miR-361-3p and targeting SH2B1 33. Xu et al. reported that BBOX1-AS1 upregulates HOXC6 expression through miR-361-3p and HuR to drive cervical cancer progression 34. BBOX1-AS1 was also recently demonstrated to promote the proliferation and inhibit the apoptosis of gastric cancer cells 35. However, the expression patterns and functions of BBOX1-AS1 in HCC have not been reported to date.

In the TCGA HCC cohort, we found that BBOX1-AS1 was overexpressed in cancerous tissues compared with adjacent normal tissues, and its high expression was associated with unfavorable clinical characteristics. Furthermore, we found that high BBOX1-AS1 expression was highly correlated with worse overall survival and relapse-free survival. Subsequently, the expression and function of BBOX1-AS1 in HCC was validated using clinical samples, *in vitro*, and *in vivo* experiments. Our results were consistent with the conclusions of the analysis of public databases. To our best knowledge, this is the first study to explore the expression and function of BBOX1-AS1 in HCC.

In conclusion, WGCNA was used to identify key modules related to VI and OS in HCC. We then established a VI-related prognostic lncRNA signature that may be an independent biomarker for predicting the clinical outcomes of HCC patients. Finally, we constructed a VI-related lncRNA-miRNA-mRNA ceRNA network and screened a hub lncRNA, whose role was validated by data mining and *in vitro* experiments.

## Supplementary Material

Supplementary figures and tables.

## Abbreviations

VI: vascular invasion
HCC: hepatocellular carcinoma
DEGs: differentially expressed genes
WGCNA: weighted gene co-expression network analysis
EMT: epithelial-to-mesenchymal transition

## References

[1] Erstad DJ, Tanabe KK (2019). Prognostic and Therapeutic Implications of Microvascular Invasion in Hepatocellular Carcinoma. ANN SURG ONCOL.

[2] Fei M, Guan J, Xue T (2018). Hypoxia promotes the migration and invasion of human hepatocarcinoma cells through the HIF-1alpha-IL-8-Akt axis. CELL MOL BIOL LETT.

[3] Lou W, Chen J, Ding B (2018). Identification of invasion-metastasis-associated microRNAs in hepatocellular carcinoma based on bioinformatic analysis and experimental validation. J TRANSL MED.

[4] Mittal K, Ebos J, Rini B (2014). Angiogenesisand the tumor microenvironment: vascular endothelial growth factor and beyond. SEMIN ONCOL.

[5] Zhang R, Ye J, Huang H, Du X (2019). Mining featured biomarkers associated with vascular invasion in HCC by bioinformatics analysis with TCGA RNA sequencing data. BIOMED PHARMACOTHER.

[6] Sumie S, Nakashima O, Okuda K (2014). The significance of classifying microvascular invasion in patients with hepatocellular carcinoma. ANN SURG ONCOL.

[7] Hsieh CH, Wei CK, Yin WY (2015). Vascular invasion affects survival in early hepatocellular carcinoma. Mol Clin Oncol.

[8] Li R, Wang Y, Zhang X (2019). Exosome-mediated secretion of LOXL4 promotes hepatocellular carcinoma cell invasion and metastasis. MOL CANCER.

[9] Zhang ZQ, Chen J, Huang WQ (2019). FAM134B induces tumorigenesis and epithelial-to-mesenchymal transition via Akt signaling in hepatocellular carcinoma. MOL ONCOL.

[10] Vasuri F, Visani M, Acquaviva G (2018). Role of microRNAs in the main molecular pathways of hepatocellular carcinoma. World J Gastroenterol.

[11] Li B, Mao R, Liu C (2018). LncRNA FAL1 promotes cell proliferation and migration by acting as a CeRNA of miR-1236 in hepatocellular carcinoma cells. LIFE SCI.

[12] Wang T, Xu L, Jia R, Wei J (2017). MiR-218 suppresses the metastasis and EMT of HCC cells via targeting SERBP1. Acta Biochim Biophys Sin (Shanghai).

[13] Yu M, Xue H, Wang Y (2017). miR-345 inhibits tumor metastasis and EMT by targeting IRF1-mediated mTOR/STAT3/AKT pathway in hepatocellular carcinoma. INT J ONCOL.

[14] Zhang YT, Li BP, Zhang B (2018). LncRNA SBF2-AS1 promotes hepatocellular carcinoma metastasis by regulating EMT and predicts unfavorable prognosis. Eur Rev Med Pharmacol Sci.

[15] Yuan S, Si W, Zhuang K (2021). LncRNA UCID Promotes Hepatocellular Carcinoma Metastasis via Stabilization of Snail. Onco Targets Ther.

[16] Yuan JH, Yang F, Wang F (2014). A long noncoding RNA activated by TGF-beta promotes the invasion-metastasis cascade in hepatocellular carcinoma. CANCER CELL.

[17] Han B, Zheng Y, Wang L (2019). A novel microRNA signature predicts vascular invasion in hepatocellular carcinoma. J CELL PHYSIOL.

[18] Ding S, Jin Y, Hao Q (2020). LncRNA BCYRN1/miR-490-3p/POU3F2, served as a ceRNA network, is connected with worse survival rate of hepatocellular carcinoma patients and promotes tumor cell growth and metastasis. CANCER CELL INT.

[19] Mo J, Li B, Zhou Y (2019). LINC00473 promotes hepatocellular carcinoma progression via acting as a ceRNA for microRNA-195 and increasing HMGA2 expression. BIOMED PHARMACOTHER.

[20] Long J, Bai Y, Yang X (2019). Construction and comprehensive analysis of a ceRNA network to reveal potential prognostic biomarkers for hepatocellular carcinoma. CANCER CELL INT.

[21] Bai Y, Long J, Liu Z (2019). Comprehensive analysis of a ceRNA network reveals potential prognostic cytoplasmic lncRNAs involved in HCC progression. J CELL PHYSIOL.

[22] Zhang J, Bian Z, Jin G (2019). Long non-coding RNA IQCJ-SCHIP1 antisense RNA 1 is downregulated in colorectal cancer and inhibits cell proliferation. Ann Transl Med.

[23] Song SK, Jung WY, Park SK (2019). Significantly different expression levels of microRNAs associated with vascular invasion in hepatocellular carcinoma and their prognostic significance after surgical resection. PLOS ONE.

[24] Zhang PF, Wang F, Wu J (2019). LncRNA SNHG3 induces EMT and sorafenib resistance by modulating the miR-128/CD151 pathway in hepatocellular carcinoma. J CELL PHYSIOL.

[25] Guo D, Li Y, Chen Y (2019). DANCR promotes HCC progression and regulates EMT by sponging miR-27a-3p via ROCK1/LIMK1/COFILIN1 pathway. Cell Prolif.

[26] Li W, Chen QF, Huang T (2020). Identification and Validation of a Prognostic lncRNA Signature for Hepatocellular Carcinoma. FRONT ONCOL.

[27] Yan J, Zhou C, Guo K (2019). A novel seven-lncRNA signature for prognosis prediction in hepatocellular carcinoma. J CELL BIOCHEM.

[28] Atianand MK, Hu W, Satpathy AT (2016). A Long Noncoding RNA lincRNA-EPS Acts as a Transcriptional Brake to Restrain Inflammation. CELL.

[29] Xia M, Liu J, Liu S (2017). Ash1l and lnc-Smad3 coordinate Smad3 locus accessibility to modulate iTreg polarization and T cell autoimmunity. NAT COMMUN.

[30] Ye T, Yang X, Liu H (2020). Long Non-Coding RNA BLACAT1 in Human Cancers. Onco Targets Ther.

[31] Wang QM, Lian GY, Song Y (2019). LncRNA MALAT1 promotes tumorigenesis and immune escape of diffuse large B cell lymphoma by sponging miR-195. LIFE SCI.

[32] Ye J, Zhang J, Lv Y (2019). Integrated analysis of a competing endogenous RNA network reveals key long noncoding RNAs as potential prognostic biomarkers for hepatocellular carcinoma. J CELL BIOCHEM.

[33] Liu J, Zhu J, Xiao Z (2020). BBOX1-AS1 contributes to colorectal cancer progression by sponging hsa-miR-361-3p and targeting SH2B1. FEBS OPEN BIO.

[34] Xu J, Yang B, Wang L (2020). LncRNA BBOX1-AS1 upregulates HOXC6 expression through miR-361-3p and HuR to drive cervical cancer progression. Cell Prolif.

[35] Yang Y, Yu Q, Li B (2020). BBOX1-AS1 Accelerates Gastric Cancer Proliferation by Sponging miR-3940-3p to Upregulate BIRC5 Expression. Dig Dis Sci.

