# Supplementary material for: Construction of a ceRNA Network and a Prognostic lncRNA Signature associated with Vascular Invasion in Hepatocellular Carcinoma based on Weighted Gene Co-Expression Network Analysis
Source: J Cancer. 2021 May 5;12(13):3754–68. doi: 10.7150/jca.57260 (PMC8176257; doi:10.7150/jca.57260)

Table S1. siRNA target sequences and sh-BBOX1-AS1 sequence

	Sequence
**siRNA1**	GAAGTTTCTTTCAAACTCT
**siRNA2**	TCTAAAAGCAGGCAGAGTTTG
**sh-BBOX1-AS1**	CCGCTCTAAAAGCAGGCAGAGTTTGCTCGAGCAAACTCTGCCTGCTTTTAGATTTTTG

Table S2. The sequences of primers

Gene	Forward(5' to 3')	Reverse(5' to 3')
**BBOX1 -AS1**	CCTGAATACCAAAGAGGGCCG	TGAAGCCTCTCTCTGCTAGGT
**GAPDH**	GGAGCGAGATCCCTCCAAAAT	GGCTGTTGTCATACTTCTCATGG

Table S3. K-M survival analysis results of 16 lncRNA that were significantly correlated with OS

Gene name	HR	95%CI	P value
AC004704.1	2	1.28-3.13	0.002
TMEM132D-AS1	2.7	1.69-4.3	＜0.001
LINC00559	1.79	1.14-2.79	0.01
LINC00501	3.31	2.07-5.28	＜0.001
AC022639.1	1.75	1.18-2.61	0.005
ARHGAP31-AS1	1.92	1.36-2.72	＜0.001
AP001972.4	1.86	1.32-2.63	＜0.001
AP001099.1	1.76	1.22-2.53	0.002
AL683887.1	1.91	1.26-2.87	0.002
AL445430.1	2.09	1.32-3.29	0.001
AC121342.1	2.24	1.43-3.51	＜0.001
AC114803.1	2.56	1.79-3.67	＜0.001
EGLN3-AS1	2.08	1.43-3.01	＜0.001
AC114489.1	2.5	1.61-3.87	＜0.001
AC006372.1	1.82	1.2-2.76	0.004
AC005381.1	1.59	1.12-2.24	0.008

Table S4. 62 genes significantly correlated with the OS

Ensembl ID	symbol	logFC	logCPM	LR	PValue	FDR	change
ENSG00000240990	HOXA11-AS	5.594335	5.159335	76.32536	2.41E-18	2.09E-16	UP
ENSG00000254560	BBOX1-AS1	4.541	5.676473	45.05503	1.92E-11	4.53E-10	UP
ENSG00000250451	HOXC-AS1	5.503758	4.307539	40.68347	1.79E-10	3.49E-09	UP
ENSG00000250033	SLC7A11-AS1	3.64072	4.425083	34.90664	3.46E-09	5.23E-08	UP
ENSG00000267243	AC005381.1	4.934267	4.937968	34.67332	3.90E-09	5.83E-08	UP
ENSG00000227195	MIR663AHG	6.085244	5.550596	34.04882	5.37E-09	7.85E-08	UP
ENSG00000246228	CASC8	3.343309	5.013412	33.85046	5.95E-09	8.60E-08	UP
ENSG00000224409	AC114489.2	4.45649	3.775562	31.50255	1.99E-08	2.63E-07	UP
ENSG00000229191	AL358473.1	2.384513	3.540638	29.92763	4.48E-08	5.42E-07	UP
ENSG00000249196	TMEM132D-AS1	7.262596	6.698578	29.92249	4.50E-08	5.43E-07	UP
ENSG00000243810	CFAP61-AS1	4.579912	4.164345	29.3515	6.04E-08	7.09E-07	UP
ENSG00000256732	AC006065.4	5.780465	5.915296	28.4177	9.78E-08	1.10E-06	UP
ENSG00000203645	LINC00501	2.799758	4.183812	27.26757	1.77E-07	1.90E-06	UP
ENSG00000259485	LINC02253	4.550949	5.222779	26.85333	2.19E-07	2.30E-06	UP
ENSG00000228742	LINC02577	4.461286	4.338651	25.54907	4.31E-07	4.28E-06	UP
ENSG00000263551	AP000829.1	3.169028	3.305601	24.05247	9.37E-07	8.63E-06	UP
ENSG00000233928	AL591501.1	4.216705	3.516369	23.9796	9.74E-07	8.92E-06	UP
ENSG00000262521	AJ003147.1	3.044474	3.009679	23.11309	1.53E-06	1.34E-05	UP
ENSG00000258897	EGLN3-AS1	3.967898	3.391069	22.96906	1.65E-06	1.43E-05	UP
ENSG00000274213	AC015912.3	1.083706	6.030234	22.38028	2.24E-06	1.87E-05	UP
ENSG00000231439	WASIR2	2.003585	3.654184	22.29673	2.34E-06	1.94E-05	UP
ENSG00000255395	AP001972.4	4.091786	4.92316	19.8732	8.28E-06	6.13E-05	UP
ENSG00000265728	AP001099.1	2.48646	3.774688	18.55687	1.65E-05	0.000113	UP
ENSG00000228127	LINC01649	3.395504	3.353891	18.40857	1.78E-05	0.000121	UP
ENSG00000240922	LSAMP-AS1	3.07954	3.024703	18.10596	2.09E-05	0.000139	UP
ENSG00000229404	LINC00858	3.311537	3.898431	17.78823	2.47E-05	0.000162	UP
ENSG00000228723	SRGAP3-AS2	3.538902	3.650821	17.60314	2.72E-05	0.000176	UP
ENSG00000272473	AC006273.1	1.399953	4.779207	16.925	3.89E-05	0.000242	UP
ENSG00000259514	AC027243.2	2.854145	3.050605	16.86505	4.01E-05	0.000249	UP
ENSG00000272202	AC097358.2	2.187393	3.041461	15.53576	8.10E-05	0.000463	UP
ENSG00000227088	AC012494.1	2.156036	2.77512	14.10704	0.000173	0.000905	UP
ENSG00000230432	AC114803.1	2.624059	3.05908	13.61863	0.000224	0.001142	UP
ENSG00000226900	AL451069.1	1.894448	4.503946	13.41954	0.000249	0.001256	UP
ENSG00000275512	AC007998.4	1.399956	3.029446	11.22679	0.000806	0.003533	UP
ENSG00000265843	LINC01029	4.549287	3.924673	10.86495	0.00098	0.00418	UP
ENSG00000250166	AC053513.1	2.29243	2.814284	10.36229	0.001286	0.005304	UP
ENSG00000234352	AC009264.1	2.537818	3.740365	10.35456	0.001292	0.005323	UP
ENSG00000249815	AC004704.1	2.410471	2.908076	10.0243	0.001545	0.006201	UP
ENSG00000235601	BARX1-DT	2.394762	3.094697	9.942803	0.001615	0.006441	UP
ENSG00000233403	AC121342.1	2.648944	2.905922	9.472936	0.002085	0.008009	UP
ENSG00000248973	AC106799.2	2.450594	3.024166	9.297516	0.002295	0.008672	UP
ENSG00000260763	AC106799.3	2.207494	3.162484	9.085944	0.002576	0.009617	UP

ENSG00000224228	AL031599.1	1.853727	2.978406	9.01919	0.002672	0.009918	UP
ENSG00000236289	GACAT3	2.625072	3.236905	8.906434	0.002842	0.010452	UP
ENSG00000231689	LINC01090	2.199405	3.089525	8.544965	0.003465	0.012411	UP
ENSG00000241449	AC092666.1	1.588577	2.833917	8.137833	0.004335	0.015032	UP
ENSG00000226629	LINC00974	1.858796	2.719607	7.941998	0.00483	0.016518	UP
ENSG00000082929	LINC01587	1.385419	5.95765	7.774194	0.0053	0.017898	UP
ENSG00000225334	LINC02813	1.815252	2.760121	7.549847	0.006002	0.019898	UP
ENSG00000248475	AC016642.1	2.230993	3.171897	7.516585	0.006113	0.02021	UP
ENSG00000230836	LINC01293	1.394164	3.423984	7.106748	0.007679	0.024562	UP
ENSG00000227685	LINC02088	2.18567	2.868642	6.840919	0.008909	0.027881	UP
ENSG00000253554	LINC01414	2.097577	3.116501	6.392228	0.011462	0.034602	UP
ENSG00000275016	AC015574.1	2.605171	3.295642	6.170832	0.012987	0.03843	UP
ENSG00000248112	AC108174.1	1.330509	2.772006	6.161584	0.013055	0.038609	UP
ENSG00000236648	LINC02810	2.64448	3.065298	6.052522	0.013886	0.040614	UP
ENSG00000254123	LINC02839	1.411708	2.651863	5.716878	0.016803	0.047622	UP
ENSG00000235615	AJ239322.1	1.959698	2.964588	5.687239	0.017089	0.04826	UP
ENSG00000233656	AL445430.1	1.114352	2.896433	4.970324	0.025786	0.06778	UP
ENSG00000226921	LINC00454	1.833141	2.806876	4.463222	0.034632	0.086428	UP
ENSG00000223872	AC006372.1	1.851957	3.242938	4.425924	0.035397	0.087919	UP
ENSG00000249785	LINC02103	1.128726	2.61421	3.874109	0.049036	0.115242	UP

Figure S1

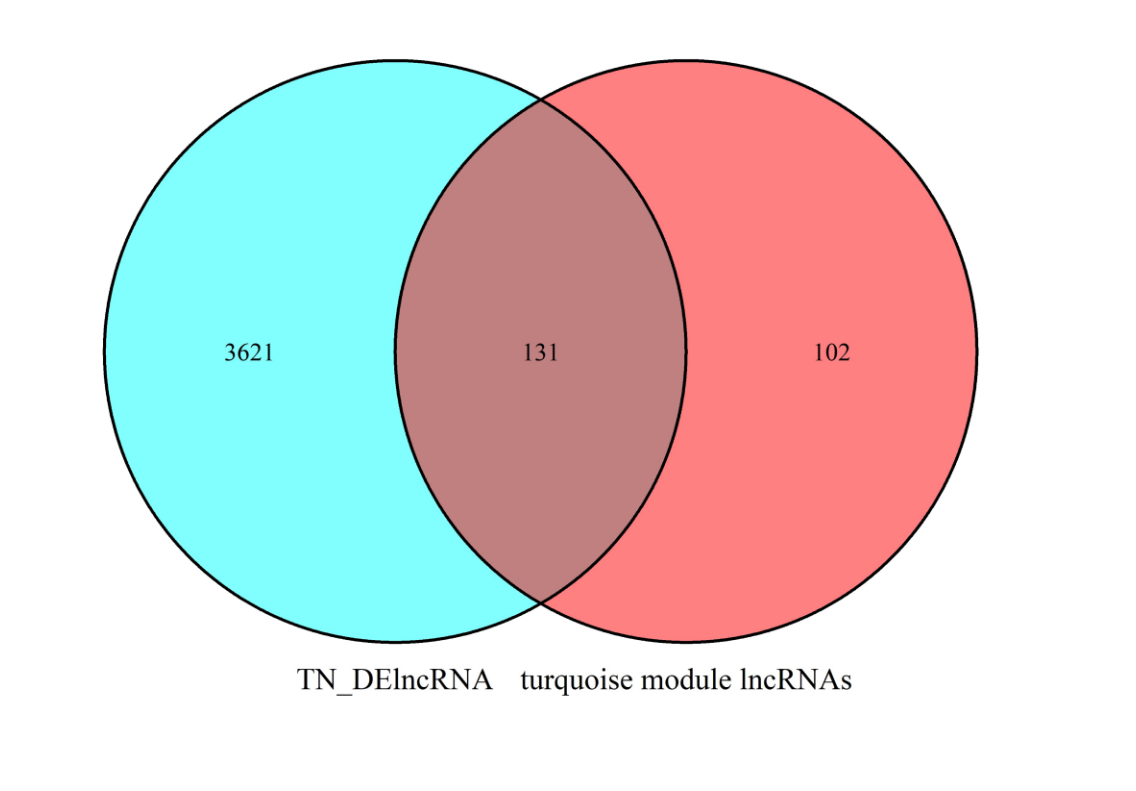

Supplement: Supplementary file 1 — Supplementary figures and tables. [file jcav12p3754s1.pdf]
